# Author Correction: Brain Data Standards - A method for building data-driven cell-type ontologies

**DOI:** 10.1038/s41597-023-02165-4

**Published:** 2023-04-28

**Authors:** Shawn Zheng Kai Tan, Huseyin Kir, Brian D. Aevermann, Tom Gillespie, Nomi Harris, Michael J. Hawrylycz, Nikolas L. Jorstad, Ed S. Lein, Nicolas Matentzoglu, Jeremy A. Miller, Tyler S. Mollenkopf, Christopher J. Mungall, Patrick L. Ray, Raymond E. A. Sanchez, Brian Staats, Jim Vermillion, Ambika Yadav, Yun Zhang, Richard H. Scheuermann, David Osumi-Sutherland

**Affiliations:** 1 European Bioinformatics Institute (EMBL-EBI), Wellcome Trust Genome Campus, Hinxton, Cambridge, United Kingdom; 2 J. Craig Venter Institute (JCVI), La Jolla, CA USA; 3 University of California San Diego, La Jolla, CA USA; 4 Lawrence Berkeley National Laboratory, Berkeley, CA USA; 5 Allen Institute for Brain Science, Seattle, WA USA; 6 Semanticly Ltd, Athens, United Kingdom

**Keywords:** Cellular neuroscience, Transcriptomics, Cellular neuroscience, Transcriptomics

Correction to: *Scientific Data* 10.1038/s41597-022-01886-2, published online 24 January 2023

In this article the funding from ‘NIMH/NIH:1U24MH114827-01 - “A Community Resource for Single Cell Data in the Brain”. ’ was omitted. The original article has been corrected.

